# Syndromes with congenital brittle bones

**DOI:** 10.1186/1471-2431-4-16

**Published:** 2004-08-31

**Authors:** Horacio Plotkin

**Affiliations:** 1 Inherited Metabolic Diseases Section, Department of Pediatrics, University of Nebraska Medical Center, And Children's Hospital, Omaha, Nebraska, USA

**Keywords:** classification, osteogenesis imperfecta, brittle bones, congenital

## Abstract

**Background:**

There is no clear definition of osteogenesis imperfecta (OI). The most widely used classification of OI divides the disease in four types, although it has been suggested that there may be at least 12 forms of OI. These forms have been named with numbers, eponyms or descriptive names. Some of these syndromes can actually be considered congenital forms of brittle bones resembling OI (SROI).

**Discussion:**

A review of different syndromes with congenital brittle bones published in the literature is presented. Syndromes are classified in "OI" (those secondary to mutations in the type I pro-collagen genes), and "syndromes resembling OI" (those secondary to mutations other that the type I pro-collagen genes, identified or not). A definition for OI is proposed as a syndrome of congenital brittle bones secondary to mutations in the genes codifying for pro-collagen genes (COL1A1 and COL1A2).

**Summary:**

A debate about the definition of OI and a possible clinical and prognostic classification are warranted.

## Background

Besides brittle bones, all other clinical characteristics of osteogenesis imperfecta (OI) are variable, and even different members of the same family may present with a dissimilar degree of severity [1]. Severity appears to follow a continuum in the OI population, and it is therefore very difficult to establish a clinical prognostic classification in definite categories. This disease has received different names through history, including osteopsathyrosis, Vrolik's disease, fragilitas ossium, mollities ossium, Lobstein's disease, and Van der Hoeve syndrome [2]. In a first attempt to classify OI, in 1906 Looser divided the disease in two forms [3]: "congenita" (Vrolik) and "tarda" (Lobstein), depending on the severity of the presentation. In OI congenita, multiple fractures may occur *in-utero*, whereas in OI tarda, fractures happen at the time of birth or later. OI tarda has also been sub-divided in "gravis" and "levis" [3]. This classification is no longer tenable because it failed to encompass the obvious clinical variability apparent in this disorder. There has also been an attempt to classify OI according to radiological characteristics [4]. Some of the features suggested by the authors of this classification are not present until age 5 or 10 years, and children of early ages can not be classified using this scheme. There has also been suggested that OI could be classified OI according to severity [5,6]. The most commonly used classification, initially proposed by Sillence in 1979, divides the patients in four types (I-IV) [7,8]. In fact, it is now widely recognized that there may be more types of OI [9-11]. Some of the forms of congenital brittle bones have been considered OI and have been added as numbers V, VI and VII [8,12-14], while others have been designated with eponyms (Cole-Carpenter syndrome [15], Bruck syndrome [16]), and others designated with clinical features (OI with denser areas in bones [17], OI with optic atrophy, retinopathy, and severe psychomotor retardation [18], OI with microcephaly, and cataracts [19], osteoporosis-pseudoglioma syndrome). The numeric classification (I-VII) is somewhat confusing, as the characteristics of each type overlap. Furthermore, there is no consensus about basic characteristics of the different types. For example, it is not clear if type I OI includes individuals with short stature, or if bone deformity rules out the diagnosis of Type I OI. Also, it is unclear if an individual with normal height can have type IV OI. Type II OI is defined as lethal, but there are cases of children with clinical characteristics of type II OI who have survived several years. Furthermore, the current classification does not allow for prognostic predictions, as individuals with type I OI may have numerous fractures and chronic pain in the course of their lives. This is part of the lack of a precise definition of OI. Thus, despite the fact that OI has been known for more than 200 years, there is no consensus about the definition for the disease. For some, it includes only those forms of congenital brittle bones secondary to mutations in the genes codifying for type I pro-collagen (COL1A1 and COL1A2). For others, it is a group of conditions with the common feature of congenital brittle bones. The example of the osteogenesis imperfecta-pseudoglioma syndrome illustrates this concept very clearly. This syndrome has been re-named as "osteoporosis-pseudoglioma syndrome" once the causal mutation has been identified to be in the LRP5 gene, elsewhere than the type I pro-collagen genes [20,21]. Following this example, I propose to define osteogenesis imperfecta as syndromes resulting from mutations in either COL1A1 or COL1A2 genes, and to group all other syndromes with congenital brittle bones as "syndromes resembling OI" (SROI), pending the identification of their causal mutation. Here, a review of the different forms of syndromes with congenital brittle bones is presented (Table 1). A working-group of international experts (like the Villefrance criteria in Ehlers-Danlos Syndrome) to clarify the issue of definition of congenital brittle bones syndromes and their classification is warranted.

**Table 1:** syndromes with congenital brittle bones

Osteogenesis Imperfecta
Mild OI with normal stature
Moderate OI with short stature
Severe OI
Lethal OI
Congenital brittle bones with dense areas in bones
Syndromes resembling OI (SROI)
Congenital brittle bones with craniosynostosis and ocular proptosis
Congenital brittle bones with congenital joint contractures
Osteoporosis-pseudoglioma syndrome
Congenital brittle bones with optic atrophy, retinopathy and severe psychomotor retardation
Congenital brittle bones with microcephaly and cataracts
Congenital brittle bones with redundant callus
Congenital brittle bones with mineralization defect
Congenital brittle bones with rhizomelia

## Discussion

Here, syndromes with congenital brittle bones are divided in osteogenesis imperfecta (those secondary to mutations in the type I pro-collagen genes) and syndromes resembling OI (those secondary to mutations other than the type I pro-collagen genes, identified or not)

## Osteogenesis imperfecta (types are ordered based on severity from mild to severe)

### Type I OI

The most common mutation causing OI type I causes a reduction in the production of otherwise normal type I collagen secondary to the effect of a null allele mutation. Patients often have normal stature, and a slightly low stature does not preclude the diagnosis of type I OI. "Type I OI" is not a synonymous of "mild OI". Individuals may have few or no fractures, mostly during the first years of life or even at birth [22], or numerous fractures throughout their lives. They may have triangular face. They are fully ambulatory, and do not have bowing of the long bones, although vertebral fractures may be present. Most have blue sclera, but it can be white, or blue color may fade as the individual grows older. This condition is transmitted as an autosomal dominant trait. Despite the absence of fractures, bone density can be very low, with no relation with clinical severity, underscoring the relative lack of significance of bone density measurements assessing severity in patients with OI. In many instances bone density is normal during the first months of life, and individuals progressively fail to increase bone mineral density with age. In some cases the diagnosis is an incidental finding after a fracture [23]. Dentinogenesis imperfecta can be present even in very mild cases. Early hypoacusia [24,25] and cardio-vascular problems, particularly aortic valvular disease [22] can be present in these subjects.

### Type IV OI (Moderate OI with short stature)

These individuals typically have short stature, bowing of long bones, and vertebral fractures. Scoliosis and joint laxity may be present. Patients with this form of OI are generally able to ambulate, but they may require aids for walking. Based in the presence of DI, moderate OI has been sub-divided in two forms, "a" and "b" [26]. These patients have white sclera. Precise diagnosis of this type of OI is often difficult, as the clinical characteristics are not clear in the literature, and different centers base the diagnosis on different criteria.

### Type III OI (Severe OI)

These patients have triangular face, product of an enlarged head and under-development of the facial bones. They also have chest deformities, markedly short stature, and severe deformities of the long bones, vertebral fractures, and scoliosis. They are frequently wheelchair-bound, although some are able to walk with aids. Prenatal diagnosis is hard but sometimes possible using ultrasonography [27]. Long bones are severely bowed, and altered structure of the growth plates lead to a particular structural alteration of the metaphyses and epiphyses described a "popcorn appearance". Severe cases may have respiratory complications that can compromise survival.

### Type II OI

In this form of OI, most newborns do not survive the perinatal period. Causes of death are malformations or hemorrhages of the central nervous system [28], extreme fragility of the ribs, or pulmonary hypoplasia [29]. The infants present with multiple intra-uterine fractures, including skull, long bones and vertebrae, beaded ribs, and severe deformity of the long bones [30]. Prenatal differential diagnosis between severe and lethal OI is usually not possible. Extremely severe cases can be dismembered during delivery [31]. The vast majority of cases are autosomal dominant new mutations [32-34]. It has been suggested that there may be different clinical forms of lethal OI [35]. Despite severity, a few patients have survived for several years.

### Congenital brittle bones with dense areas in bones

Described in one infant [17] who died shortly after birth and presented with an OI phenotype that differed from the usual lethal form. The skeleton had regions of increased bone density, and this girl had dysmorphic facial features, including loss of mandibular angle, low set ears, soft skull, and large anterior and posterior fontanelles. Bilateral upper and lower limb contractures were present with multiple fractures in the long bones and ribs. The metaphysis of the long bones were dense in x-rays. The patient died after a few hours and histopathological studies identified extramedullary hematopoiesis in the liver, little lamellar bone formation, decreased number of osteoclasts, abnormally thickened bony trabeculae with retained cartilage in long bones, and diminished marrow spaces similar to those seen in dense bone diseases such as osteopetrosis and pycnodysostosis. Genetic testing showed that the child was heterozygous for a COL1A14321G → T transversion in exon 52 that changed a conserved aspartic acid to tyrosine (D1441Y). Abnormal proa1(I) chains were slow to assemble into dimers and trimers, and abnormal molecules were retained intracellularly for an extended period [17]. Because of this mutation, this form of congenital brittle bones should be included in the OI group.

### Syndromes resembling OI (SROI)

#### Congenital brittle bones with craniosynostosis and ocular proptosis (Cole-Carpenter syndrome)

Two boys [15] and a girl [36] with this particular form of SROI have been described in the literature. Both boys were normal at birth, but after several months, they developed multiple metaphyseal fractures, associated with low bone density in the entire skeleton and craniosynostosis, hydrocephalus, ocular proptosis, and facial dysmorphism. One of the patients had also hypercalciuria. Neurological development is normal in this form of SROI. Both patients where wheelchair-bound at adult age, with very short stature, severe bone involvement and normal intellectual and neurological development [10]. No mutation has been identified as causing this syndrome.

#### Congenital brittle bones with congenital joint contractures (Bruck syndrome)

First described by Bruck et al in 1897 in an adult patient [16], in this form of SROI patients are born with brittle bones, leading to multiple fractures and joint contractures and pterygia (arthrogryposis multiplex congenita) due to dislocation of the radial head [37,38]. Wormian bones are present, and inheritance appears to be recessive [39,40]. In three patients that underwent pro-collagen mutation testing, it was not possible to demonstrate any mutations in the COL1A1 and COL1A2 genes [38]. The basic defect was mapped to locus 17p12 (18 cM interval), where a bone telopeptidyl hydroxylase is located [41]. The mutation leads to underhydroxilated lysine residues within the telopeptides of collagen type I, and therefore to aberrant crosslinking in bone, but not in cartilage or ligaments.

#### Osteoporosis pseudoglioma syndrome [20,21]

This form of SROI was first described in three families in 1972 [42]. Other report described a South African family of Indian origin with the condition [43]. Inheritance is autosomal recessive. Individuals with Osteoporosis-pseudoglioma syndrome have mild to moderate OI with blindness due to hyperplasia of the vitreous, corneal opacity and secondary glaucoma. The ocular pathology may be secondary to failed regression of the primary vitreal vasculature during fetal growth [44]. The genetic defect has been mapped to chromosome region 11q12-13 [45]. The defect is specifically in the LRP5 gene that encodes for the low-density lipoprotein receptor-related protein 5 [44]. Treatment with pamidronate has shown promising results in this group of patients [46].

#### Other forms of SROI with ocular involvement

Two other forms of SROI with ocular involvement have been described: one variant with optic atrophy, retinopathy, and severe psychomotor retardation [18], another with microcephaly and cataracts [19].

#### Congenital brittle bones with redundant callus formation

Originally published as "type V OI" [12]. Patients with this syndrome develop hyperplastic calluses in long bones after a fracture or intramedullary rodding surgery [22]. These patients present with hard, painful and warm swellings over long bones which initially may suggest inflammation or osteosarcoma. After a rapid growth period, the size and shape of the callus may remain stable for many years [47]. Microscopically there is increased production of abnormal extracellular matrix, that is poorly organized and incompletely mineralized in the callus area [48]. Histological studies in bone outside the callus area showed that the bone lamellae are arranged in a mesh-like fashion, as opposed to a parallel arrangement in patients with OI [12]. A series of case reports of hyperplastic callus formation in patients with clinical characteristics compatible with OI can be found in the literature [3,47,49-54]. These large calluses may also be present in flat bones [55].

These patients may also have calcification of the interosseous membrane between the radius and ulna, determining a clinical sign, as affected individuals are unable to pronate and supinate the forearm. The radial head may be dislocated. Patients with this syndrome have white sclera and no DI. Mutations in the pro-collagen genes could not be identified so far. Inheritance appears to be autosomal dominant, with variable penetrance.

#### Congenital brittle bones with mineralization defect

Undistinguishable from moderate to severe OI on a clinical basis, this rare form of SROI [13], has been classified as "Type VI OI". It can only be diagnosed by bone biopsy, where a mineralization defect affecting the bone matrix and sparing growth cartilage is evident. These patients have no DI and no wormian bones. Despite the histological mineralization defect, there are no radiological signs of growth plate involvement. The pattern of inheritance is not clear, but the case of two siblings from healthy consanguineous parents has been described, suggesting gonadal mosaicism or a somatic recessive trait [13]. No mutations of COL1A1 and COL1A2 genes have been found in these patients, and collagen structure appears to be normal. This form of SROI shares several characteristics with fibrogenesis imperfecta ossium [56,57].

#### Congenital brittle bones with rhizomelia

A particular form of SROI with short humerus and recessive inheritance was described in a First Nations community of Quebec, and published as "type VII OI" [14]. The individuals affected have short humeri and femora. The phenotype is clinically moderate to severe. Fractures may be present at birth, and the condition progresses to early lower limb deformities, coxa vara and osteopenia. The bone morphology in congenital brittle bones with rhizomelia is not different than that of mild OI by histomorphometry. The genetic defect has been mapped to the short arm of chromosome 3 by linkage studies [58], where there are no genes that codify for type I pro collagen.

## Summary

A definition for osteogenesis imperfecta is proposed based on the presence of type I pro-collagen mutations. Inclusion of related syndromes with unidentified mutations should be considered as SROI until further defined. Different forms of syndromes with congenital brittle bones are reviewed. A debate about the definition of OI and a possible clinical and prognostic classification are warranted.

## List of abbreviations

OI Osteogenesis Imperfecta

SROI syndromes resembling osteogenesis imperfecta

DI Dentinogenesis imperfecta

## Competing interests

None declared.

## Pre-publication history

The pre-publication history for this paper can be accessed here:


